# Gene Set of Nuclear-Encoded Mitochondrial Regulators Is Enriched for Common Inherited Variation in Obesity

**DOI:** 10.1371/journal.pone.0055884

**Published:** 2013-02-08

**Authors:** Nadja Knoll, Ivonne Jarick, Anna-Lena Volckmar, Martin Klingenspor, Thomas Illig, Harald Grallert, Christian Gieger, Heinz-Erich Wichmann, Annette Peters, Johannes Hebebrand, André Scherag, Anke Hinney

**Affiliations:** 1 Department of Child and Adolescent Psychiatry, University of Duisburg-Essen, Essen, Germany; 2 Institute of Medical Biometry and Epidemiology, Philipps-University of Marburg, Marburg, Germany; 3 Molecular Nutritional Medicine, Technical University of Munich, Else Kröner-Fresenius Center, Freising-Weihenstephan, Germany; 4 Research Unit of Molecular Epidemiology, Helmholtz Center Munich – German Research Center for Environmental Health, Neuherberg, Germany; 5 Hannover Unified Biobank, Hannover Medical School, Hannover, Germany; 6 Institute of Genetic Epidemiology, Helmholtz Center Munich – German Research Center for Environmental Health, Neuherberg, Germany; 7 Institute of Epidemiology I, Helmholtz Center Munich – German Research Center for Environmental Health, Neuherberg, Germany, Neuherberg, Germany; 8 Institute of Medical Informatics, Biometry, and Epidemiology, Chair of Epidemiology, Ludwig-Maximilians-Universität, Munich, Germany; 9 Munich University Hospital, Campus Grosshadern, Munich, Germany; 10 Institute of Epidemiology II, Helmholtz Center Munich – German Research Center for Environmental Health, Neuherberg, Germany; 11 Institute for Medical Informatics, Biometry and Epidemiology, University of Duisburg-Essen, Essen, Germany; University of Tampere, Finland

## Abstract

There are hints of an altered mitochondrial function in obesity. Nuclear-encoded genes are relevant for mitochondrial function (3 gene sets of known relevant pathways: (1) 16 nuclear regulators of mitochondrial genes, (2) 91 genes for oxidative phosphorylation and (3) 966 nuclear-encoded mitochondrial genes). Gene set enrichment analysis (GSEA) showed no association with type 2 diabetes mellitus in these gene sets. Here we performed a GSEA for the same gene sets for obesity. Genome wide association study (GWAS) data from a case-control approach on 453 extremely obese children and adolescents and 435 lean adult controls were used for GSEA. For independent confirmation, we analyzed 705 obesity GWAS trios (extremely obese child and both biological parents) and a population-based GWAS sample (KORA F4, n = 1,743). A meta-analysis was performed on all three samples. In each sample, the distribution of significance levels between the respective gene set and those of all genes was compared using the leading-edge-fraction-comparison test (cut-offs between the 50^th^ and 95^th^ percentile of the set of all gene-wise corrected p-values) as implemented in the MAGENTA software. In the case-control sample, significant enrichment of associations with obesity was observed above the 50^th^ percentile for the set of the 16 nuclear regulators of mitochondrial genes (p^GSEA,50^ = 0.0103). This finding was not confirmed in the trios (p^GSEA,50^ = 0.5991), but in KORA (p^GSEA,50^ = 0.0398). The meta-analysis again indicated a trend for enrichment (p^MAGENTA,50^ = 0.1052, p^MAGENTA,75^ = 0.0251). The GSEA revealed that weak association signals for obesity might be enriched in the gene set of 16 nuclear regulators of mitochondrial genes.

## Introduction

Heritability estimates for variance of the body mass index (BMI) range between 40 to 70% [1]. The latest analysis of nearly 250,000 individuals confirmed or newly identified 32 polygenic loci that are associated with BMI [2]. These variants, however, only explain about 1.5% of the BMI variance. Because of the polygenic nature and the small effect sizes of these loci [1], [2], an estimation revealed that even an increase to a sample size of 730,000 individuals would not lead to a substantial increase in the explained BMI variance (<5%).

To discover further genetic variation associated with a certain trait, single-locus-oriented genome-wide association studies (GWAS) should be extended to pathway-based approaches or gene set enrichment analyses (GSEA). As these analyses concentrate on the combined effects across several loci, a gain in statistical power is expected and new genetic insight in the trait of interest might be revealed [3]–[5]. For example, Lui et al. [6] performed a pathway-based GWA analysis for BMI and body fat mass in US whites and showed the vasoactive intestinal peptide (VIP) pathway to be significantly associated with the investigated traits. This finding was recently confirmed by Evangelou et al. [7] in a subsample of the EPIC-Norfolk study [8].

It is well known that mitochondria are the cellular power plants whose main function is ATP production via oxidative phosphorylation (OXPHOS). Impairment of mitochondrial function might influence body weight. Indeed, there are hints that mitochondria (size, number) and mitochondrial function are altered in obesity. Adult obese individuals showed smaller mitochondria in skeletal muscle tissue [9] and a reduced complex I activity in both skeletal muscle and cardiomyocytes [9], [10] compared to lean subjects. Obesity was also associated with a reduced mRNA expression of the mitochondrial transcription factor A (Tfam) and the nuclear respiratory factor-1 (NRF1), which are two key regulators for mitochondrial biogenesis, and with strongly reduced protein and mRNA expressions of NADH dehydrogenase 6 (ND6), a subunit of complex I in the respiratory chain [10].

The mitochondrial DNA codes only for 37 genes, of which 22 encode tRNAs, two rRNAs and 13 OXPHOS subunits of the complexes I, III, IV and V. Additionally, more than 1,000 nuclear-encoded mitochondrial genes are necessary to ensure complete mitochondrial function and biogenesis [11]–[14].

Segrè et al. [15] performed a GSEA on nuclear-encoded mitochondrial genes for association with type 2 diabetes mellitus (T2DM) or related glycemic traits, and grouped these genes into three sets: (1) 16 autosomal nuclear regulators of mitochondrial genes based on the literature [12], [16]–[20]; (2) 91 autosomal OXPHOS genes [15], [21]; and (3) 966 autosomal nuclear-encoded human mitochondrial genes taken from the MitoCarta compendium [13]. Segrè et al. [15] did not find enrichment in any of the three gene sets in association with T2DM or related glycemic traits.

Based on the central role of mitochondria in metabolism and findings of an altered mitochondrial function we performed a GSEA focusing on obesity to address the question if gene sets of nuclear encoded mitochondrial genes are enriched for modest association signals that collectively influence obesity risk.

## Materials and Methods

### Study Subjects

#### Ethics Statement

Written informed consent was given by all participants and in case of minors by their parents. The study was approved by the Ethics Committees of the Universities of Marburg and Essen and the Bavarian Medical Association. It was conducted in accordance with the Declaration of Helsinki.

#### Discovery

The initial GSEA was performed in a case-control GWAS sample comprising 453 (extremely) obese children and adolescents and 435 normal weight or lean adult controls [22]. Using lean adults who were never overweight or obese during childhood (as assessed by interview) as control group reduces the chances of misclassification compared to the use of lean children as controls who might become overweight or obese in adulthood [23]. The measured body mass index (BMI; in kg/m^2^) was assessed for extremeness using age- and sex-specific percentile criteria for the German population from the National Nutrition Survey I [24]. According to this reference population, all cases were at least overweight (BMI ≥90^th^ percentile), and 84.4% were extremely obese (BMI ≥99^th^ percentile), the lean controls had a BMI of 18.31±1.11 kg/m^2^ (Table S1).

#### Confirmation

For independent confirmation a family-based GWAS sample which consisted of 705 obesity trios, each comprising one (extremely) obese child or adolescent (index case) and both biological parents, was used [22]. All index cases were at least overweight (BMI ≥90^th^ percentile), and 83.8% were extremely obese (BMI ≥99^th^ percentile) with regard to reference data from the German National Nutrition Survey I ([24]; Table S1).

In addition, we analyzed a population-based sample comprising 1,743 adult participants, which is a sub-sample of the total KORA F4 sample (Cooperative Health Research in the Region of Augsburg, Table S1, [25]). This sample was analyzed as case-control sample (KORA-CC): all individuals with a BMI ≥30 kg/m^2^ were categorized as obese cases (n  = 463) and those with a BMI <25 kg/m^2^ were coded as normal weight controls (n  = 483). This population-based sample was converted into a case-control sample, as it was recently shown that genetic markers with an effect in the extremes of a trait are detected more solidly within a case-control design compared to a linear regression design, even for smaller sample sizes [26]. This is due to the fact that linear regression results are mostly influenced by the majority of individuals with a moderate trait and only little by the few individuals of the extremes [26].

### Meta-analysis

We performed a meta-analysis of all three samples using the METAL software package (www.sph.umich.edu/csg/abecasis/metal). In more detail, meta-analysis was carried out using the inverse variance method by assuming a fixed effect model. We adopted the method of Kazeem and Farrell [27] to meta-analyze single marker information from the two case-control analyses and from the family-based TDTs.

### Genotyping

All three samples were genotyped by the Affymetrix Genome-Wide Human SNP Array 6.0. For quality control, only individuals with a genome-wide SNP call rate (CR) ≥95% were included. Furthermore, in each of the 705 trios from the family-based GWAS sample the percentage of markers with observed Mendelian inconsistent genotype calls (genome-wide 906,703 SNPs) was below 5%. Focusing on the approximate 870,000 autosomal SNPs, the following genotyping quality control filters were applied to each sample separately (Table S2): (1) sample CR ≥95%; (2) MAF ≥1% in the whole sample (case-control and population-based sample) and MAF ≥5% in the set of all parents of the family-based sample, respectively; (3) two-sided exact p-value ≥0.001 of the test for Hardy-Weinberg-Equilibrium (HWE) [28] in the whole KORA sample, in the parents of the family-based sample and in the controls of the case-controls GWAS sample, respectively. Additionally, after setting all Mendelian inconsistent calls to “missing” in the family-based sample, we claimed (4) at least one major allele and one minor allele transmission at each SNP. 703,015/641,991/659,502 autosomal SNPs passed this QC in the case-control/family-based/population-based sample, respectively and were used for the analysis (Table S2).

### Statistical Analyses

#### Gene set enrichment analysis (GSEA) for sets of mitochondrial genes

Each gene set enrichment analysis (GSEA) is based on the idea of comparing gene association signals of gene sets with biological plausibility for the given phenotype to those of the genome-wide set of genes [3], [4], [15]. First, the analyzed SNPs are linked to their corresponding gene. Secondly, a test statistic (e.g. p-value) aggregating the SNP information is derived for each gene. Finally, the distribution of test statistics is compared between gene sets (details see below).

We tested those three mitochondrial gene sets for enrichment of obesity association signals, which were considered in the study of Segrè et al. [15] who tested for an enrichment of association signals with T2DM and related glycemic traits: (1) a set of 16 autosomal nuclear regulators of mitochondrial genes based on the literature [12], [16]–[20], (2) a set of 91 autosomal oxidative phosphorylation (OXPHOS) genes [15], [21], and (3) a list of 966 autosomal nuclear-encoded human mitochondrial genes taken from the MitoCarta compendium which are over 80% of all assumed mitochondrial genes [13].

#### GSEA – Discovery

In the case-control GWAS sample, the Cochran-Armitage trend test for an additive mode of inheritance was applied to each autosomal SNP. Afterwards, SNPs were mapped onto genes. For this purpose, a list of human gene transcripts (n = 26,914 for the hg18 March 2006 version) was downloaded from the UCSC Genome Browser (http://genome.uscs.edu/). After exclusion of genes with two or more transcripts on separate chromosomes or with more than 1 Mb distance on the same chromosome, a total of 17,680 autosomal genes were followed-up. In consistency with Segrè et al. [15], SNPs that are located within 110 kb upstream and 40 kb downstream to the most extreme transcript start and end site of a gene were assigned to this gene. These boundaries were chosen as they represent the 99^th^ percentiles of the distances of cis-eQTLs from transcript start and end sites of adjacent genes [29]. Genes without SNPs in their extended gene boundaries (n = 55) were discarded from GSEA testing.

Subsequently, each gene was assigned a gene-wise empirically corrected p-value P_g_. To determine P_g_, the lowest observed p-value P_g;min_ of each gene was first determined and stored. Secondly, 10,000 permutations of the genotype data were performed using PLINK. In each permutation, affection status was flipped for all SNPs to generate the null distribution. Finally, P_g_ was calculated as the fraction of permutations whose minimal p-value per gene was equal to or smaller than P_g;min_. To achieve maximal accuracy, for those genes with P_g_ ≤0.01 (0.001), the procedure was repeated with 100,000 (1,000,000) permutations.

Prior to GSEA testing, we addressed physical clustering of genes by excluding all genes with the identical selected SNP as compared to the gene with the lowest P_g_ for the SNP already in the gene set. This exclusion was done in order to avoid significant gene set enrichment based on identical association signals [15].

Our alternative hypothesis was that gene p-value ranks in one of the three gene subsets of interest were skewed towards high ranks compared to the full autosomal set of genes. To test this hypothesis, we applied: (1) the leading-edge-fraction-comparison test as proposed by Segrè et al. [15] with P^cut-off^  = 95^th^, 75^th^ and 50^th^ percentile of the set of autosomal gene-wise p-values and with 10,000 samplings from the full autosomal gene p-value distribution (with corresponding GSEA p-values: P^GSEA,95^, P^GSEA,75^ and P^GSEA,50^). This test is based on the idea of comparing the fraction of genes with gene p-values below a certain cut-off (i.e. above a certain percentile; leading edge fraction) in the full set of genes and in the gene subset of interest. Here, the null distribution of such fractions in the gene subset is derived by randomly sampling the same number of gene p-values from the full set of gene p-values. The GSEA p-value is then determined by dividing the number of samplings with equal or larger leading edge fraction as the observed one by the number of samplings generated. To test the robustness of this test, we additionally ran three alternative one-sided GSEA tests as proposed by Segrè et al. [15]: (2) the Wilcoxon-Mann-Whitney test (P^GSEA,WMW^); (3) the Kolmogorov-Smirnov test (P^GSEA,KS^); and (4) the t-test (P^GSEA,t^).

#### GSEA – Confirmation

In the family-based GWAS sample, for each SNP a transmission disequilibrium test (TDT; [30]) was calculated using the PLINK v1.07 software [31] (http://pngu.mgh.harvard.edu/purcell/plink/) and assuming an additive allelic model of inheritance. Families with missing genotypes were excluded from TDT analysis of the respective SNP. In total, for only 0.37% of all SNPs more than 5% of trios were excluded from the TDT (due to genotyping failures and/or Mendelian inconsistencies). Gene-wise empirically corrected p-values for the family-based GWAS sample were based on randomly flipping the parentally transmitted allele for each family and each permutation. In KORA-CC each SNP was tested by the Cochran-Armitage trend test for an association with obesity, and gene-wise p-values were determined as for the discovery CC sample. For both the family-based GWAS sample and KORA-CC the remaining GSEA procedure was performed as described for the discovery sample.

#### Meta-Analysis Gene set Enrichment of variant Associations (MAGENTA)

The Meta-Analysis Gene set Enrichment of variant Associations (MAGENTA) software provided by Segrè et al. [15] was specifically designed for the application to large-genome-wide association study meta-analyses in which individual genotypes are not available. In this context, it is not possible to evaluate statistical gene-wise significance via standard phenotype permutation procedures as described above. Instead, a linear regression-based approach accounting for physical gene size, the number of SNPs and their genetic properties (LD between SNPs, number of recombination hotspots and genetic distance of the gene), was proposed to determine gene-wise corrected p-values.

After bringing together the single marker information from the two case-control samples and the family-based TDTs (in application of the METAL software; for details see ‘Meta-analysis’), we applied MAGENTA to the single marker p-values of the meta-analysis. Leading edge fraction tests for the 95^th^, the 75^th^ and the 50^th^ percentile cut-off were performed as well as the alternatively included exact Wilcoxon-Mann-Whitney test. These cut-offs were chosen because simulations showed that for modest effects the 95^th^ percentile and for weak effects the 75^th^ percentile yielded the optimal power to detect gene set enrichment [15] and with additional regard to our discovery findings.

For reasons of comparability, in addition to our permutation-based GSEA testing procedure, we applied MAGENTA to all three samples individually. Regression-corrected gene p-values (p^MAGENTA^) and permutation-based gene p-values (p^GSEA^) were shown to be highly correlated (r  = 0.95; see Table 1 & 2).

**Table 1:** Discovery: GSEA and MAGENTA for obesity in the case-control GWAS sample of 453 (extremely) obese cases and 435 lean controls.

Gene set	total number of genes	Effective number of genes	number of SNPs involved	% of all autosomal SNPs (703,015) involved	P^GSEA,WMW^, Wilcoxon-Mann-Whitneytest	P^GSEA,KS^, Kolmogorov-Smirnov-Test	P^GSEA,t^,t-Test	P^GSEA,95^, 95^th^ percentile cut-off test a	P^GSEA,75^, 75^th^ percentile cut-off test b	P^GSEA,50^, 50^th^ percentile cut-off test c	P^MAGENTA,WMW^, Wilcoxon-Mann-Whitney test d	P^MAGENTA,95^, 95^th^ percentile cut-off test a	P^MAGENTA,75^, 75^th^ percentile cut-off test b	P^MAGENTA,50^, 50^th^ percentile cut-off test c
1) Nuclear regulators of mitochondrial genes	16	16	1,014	0.14	**0.0075**	**0.0195**	**0.0053**	0.5644	0.0796	**0.0103**	**0.0043**	0.575	**0.0074**	**0.0099**
2) Oxidative phosporylation genes	91	89	2,781	0.39	0.6225	0.8586	0.6374	0.2873	0.5643	0.5834	0.8447	0.6565	0.7495	0.7369
3) Nuclear-encoded mitochondrial genes	966	880	35,223	4.93	0.3841	0.2502	0.4104	0.6437	0.1905	0.1196	0.8969	0.5287	0.7372	0.7577
all autosomal genes	17,680	10,180	521,469	73.03	reference	reference	reference	reference	reference	reference	reference	reference	reference	reference

a cut-off  = 0.0216,

b cut-off  = 0.1631,

c cut-off  = 0.3951,

d exact GSEA Wilcoxon-Mann-Whitney test; GSEA and MAGENTA p-values below 0.05 are highlighted in bold.

**Table 2:** Confirmation & Meta-analysis: GSEA and MAGENTA for the gene set of 16 nuclear regulators of mitochondrial genes in 705 trios, KORA-CC and for meta-analysis.

Sample	total number of genes	effective number of genes	number of SNPs involved	% of all autosomal SNPs involved	P^GSEA,WMW^, Wilcoxon-Mann-Whitney test	P^GSEA,KS^, Kolmogorov-Smirnov-Test	P^GSEA,t^, t-Test	P^GSEA,95^, 95^th^ percentile cut-off test a	P^GSEA,75^, 75^th^ percentile cut-off test b	P^GSEA,50^, 50^th^ percentile cut-off test c	P^MAGENTA,WMW^, Wilcoxon-Mann-Whitney test d	P^MAGENTA,95^, 95^th^ percentile cut-off test a	P^MAGENTA,75^, 75^th^ percentile cut-off test b	P^MAGENTA,50^, 50^th^ percentile cut-off test c
705 obesity trios	16	16	919	0.14	0.7879	0.7930	0.7588	1.0000	0.3711	0.5991	0.6817	1	0.6024	0.7683
463 cases and 483 controls (KORA-CC) e	16	16	933	0.14	**0.0260**	**0.0431**	**0.0211**	**0.0432**	0.1939	**0.0398**	**0.0083**	0.1918	0.1888	**0.0405**
meta-analysis	16	16	1,036	0.14	**–**	**–**	**–**	–	**–**	–	**0.0357**	0.5587	**0.0251**	0.1052

a Trios: cut-off  = 0.0382, KORA-CC: cut-off  = 0.0486, Meta-analysis: cut-off  = 0.0443,

b Trios: cut-off  = 0.2216, KORA-CC: cut-off  = 0.2611, Meta-analysis: cut-off  = 0.2969,

c Trios: cut-off  = 0.4687, KORA-CC: cut-off  = 0.5085, Meta-analysis: cut-off  = 0.5619,

d exact GSEA Wilcoxon-Mann-Whitney test,

e BMI ≥30 (cases) vs. BMI <25 (controls); GSEA p-values below 5% are highlighted in bold.

## Results

### Discovery

In our case-control sample, the effective gene set size for the GSEA analyses of all human autosomal genes was 10,180, since 55 genes did not have any genotyped SNPs within their extended gene boundaries (110 kb upstream and 40 kb downstream to the most extreme transcript boundaries) and 7,445 genes were removed due to physical clustering (see Methods). In total, all human autosomal genes were covered by 521,469 unique SNPs (73.03% of all autosomal SNPs which can be found on the Affymetrix SNP array 6.0). Among the lists of the 16 nuclear regulators of mitochondrial genes and the 91 OXPHOS genes, all genes had SNPs in their extended boundaries. Two genes of the 91 OXPHOS genes were removed due to physical clustering. There were 1,014 unique SNPs (0.14% of all SNPs) that fell within the gene regions of the nuclear regulators of mitochondrial genes and 2,781 unique SNPs (0.39% of all SNPs) that were located within the gene regions of the OXPHOS genes. Furthermore, those 965 autosomal nuclear-encoded human mitochondrial genes that contained SNPs in their gene regions were covered by 35,223 unique SNPs (4.93% of all SNPs), whereas due to physical clustering the corresponding effective gene set size was 880.

**Figure 1:**
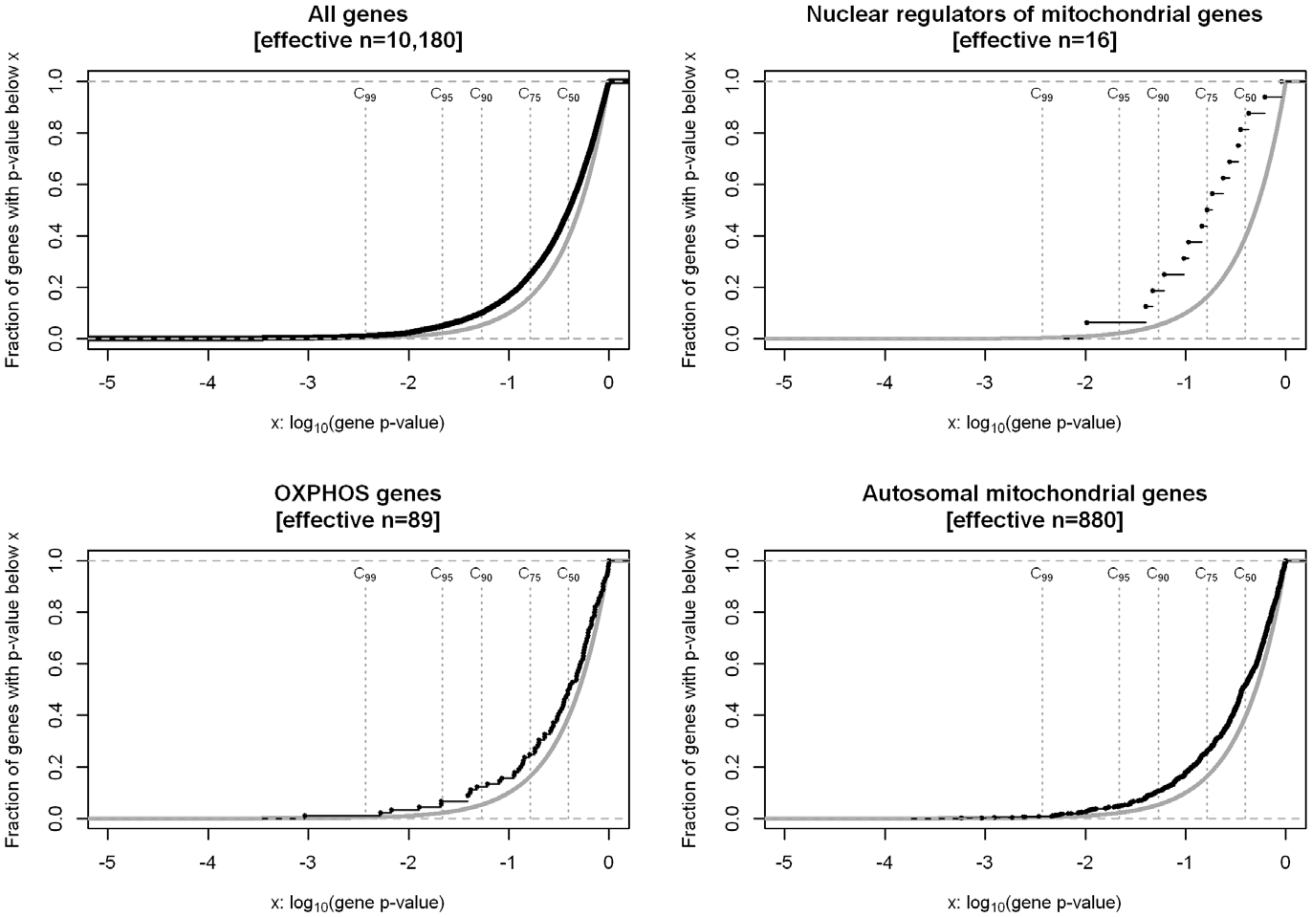
Empirical cumulative distribution functions (ECDF) of P_g_ in four different gene sets in the Discovery. A case-control GWAS sample of 453 (extremely) obese cases and 435 lean controls was analyzed. In each panel the grey line represents the ECDF of the uniform distribution (null hypotheses of no association) and the black line represents the ECDF of the respective gene set. P_g_, gene-wise corrected p-value.

The first gene set of 16 nuclear regulators of mitochondrial genes was enriched for obesity association signals (P^GSEA,WMW^  = 0.0075, P^GSEA,KS^  = 0.0195, P^GSEA,t^  = 0.0053; Fig. 1 & Table 1). This enrichment was found above the 50^th^ percentile (P^GSEA,50^ = 0.0103). The enrichment remained significant after Bonferroni correction for the three gene sets tested except for the Kolmogorov-Smirnov-Test.

### Confirmation

**Figure 2:**
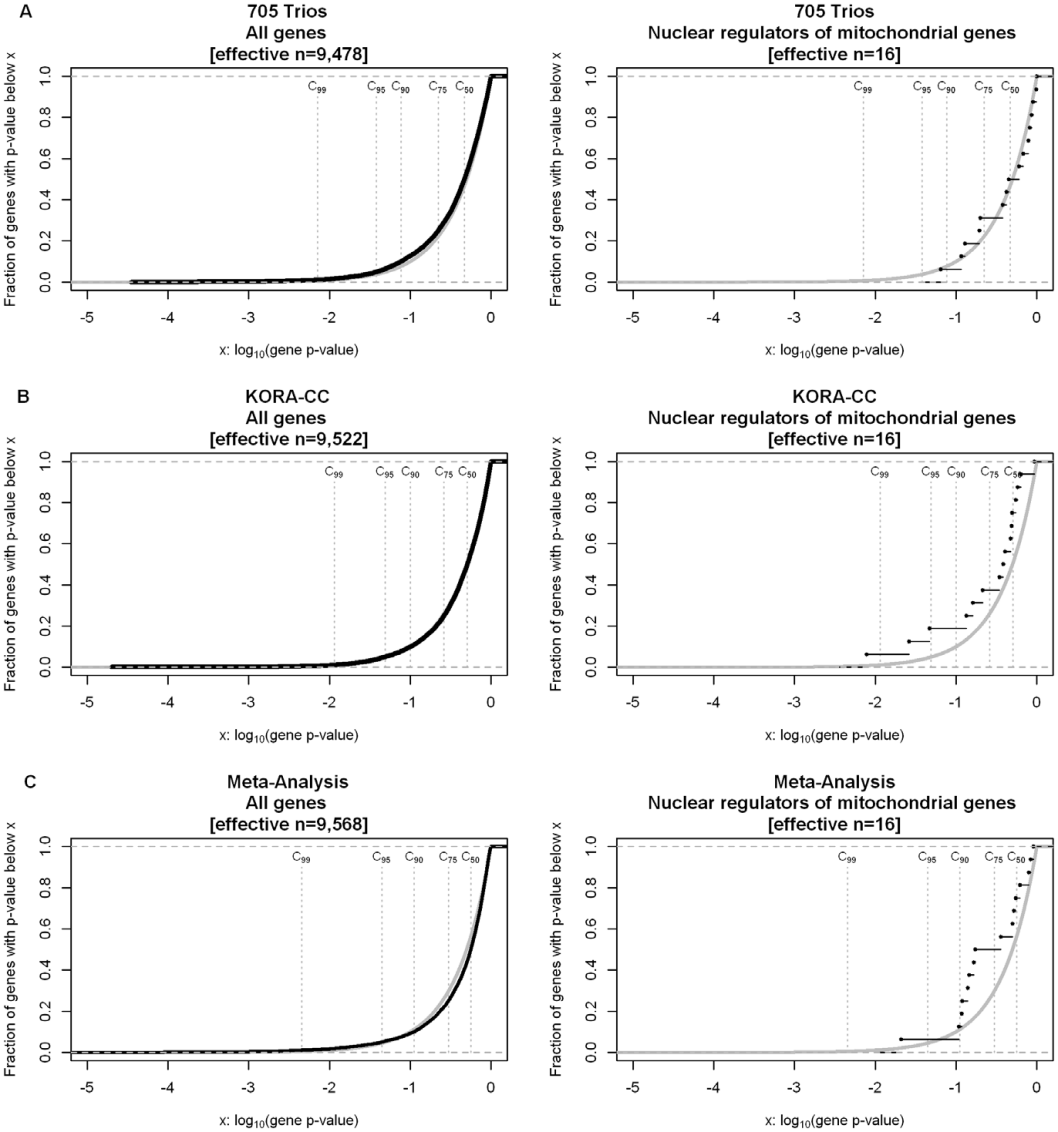
Empirical cumulative distribution functions (ECDF) of P_g_ in all autosomal genes and gene set 1. For independent confirmation of the initial finding, GSEA was performed in 705 obesity trios (**A**) and in 463 obese cases and 483 normal weight or lean controls of the KORA-CC sample (**B**). In addition, **a** meta-analysis of all three study samples (from Discovery and Confirmation) was performed (**C**). In each panel the grey line represents the ECDF of the uniform distribution (null hypotheses of no association) and the black line represents the ECDF of the respective gene set. P_g_, gene-wise corrected p-value.

In the family-based sample (705 trios), we did not observe enrichment of association signals to (early onset extreme) obesity in the first gene set (P^GSEA,50^ = 0.5991, P^GSEA,WMW^  = 0.7879, P^GSEA,KS^  = 0.7930, P^GSEA,t^  = 0.7588; Fig. 2A & Table 2).

For the second independent case-control sample (KORA-CC), we found significant enrichment for association signals for obesity for the first gene set (P^GSEA,WMW^  = 0.0260, P^GSEA,KS^  = 0.0431, P^GSEA,t^  = 0.0211). The enrichment was found above the 50^th^ percentile (P^GSEA,50^ = 0.0398), confirming the initial finding of the discovery case-control sample (Fig. 2B & Table 2).

### Meta-analysis

Due to the different design of the study samples (case-control and family-based), the permutation-based GSEA testing procedure and explicitly the determination of gene-wise corrected p-values could not directly be applied in a meta-analysis of all three samples. Alternatively, we applied the two software toolkits METAL and MAGENTA which were both specifically designed for the application to meta-analyze data. Significant enrichment for the first gene set was found (P^MAGENTA,WMW^  = 0 0357). This enrichment was not found above the 50^th^ percentile as in the discovery, but rather above the 75^th^ percentile (P^MAGENTA,50^ = 0.1052, P^MAGENTA,75^ = 0.0251; Fig. 2C & Table 2). Thus, the effect of enrichment remained stable in the meta-analysis.

For most genes, the SNP with minimal single-marker p-value differed in the three analyzed samples (Table 3). For example, rs12033461 was the best SNP for *ESRRG* in the family-based sample, while it was rs11577585 in KORA-CC. The LD between these two SNPs was r^2^ = 0 in the parents of the family-based sample. Generally, these LDs were quite low (Table 3), so association signals seem to be independent, and our gene-based approach which considers large parts of common variation within a gene might be a useful approach to handle multiple ancestral mutations [32], [33].

**Table 3:** Best SNPs of nuclear regulators of mitochondrial genes (gene set 1) in each sample and linkage disequilibrium between best SNPs of the three different study samples.

	Discovery					Confirmation				Confirmation					Meta-analysis				
Sample	453 cases and 435 controls					705 obesity trios				463 cases and 483 controls (KORA-CC) a					453 cases and 435 controls, 705 obesity trios & 463 cases and 483 controls (KORA-CC) a				
gene id	Gene p-value	Best SNP in gene b	Best SNP p-value	LD: best SNP in Trios – best SNP in CC [r^2^] c	number of SNPs in gene region	Gene p-value	Best SNP in gene b	Best SNP p-value	number of SNPs in gene region	Gene p-value	Best SNP in gene b	Best SNP p-value	LD: best SNP in Trios – best SNP in CC [r^2^] c	number of SNPs in gene region	Gene p-value	Best SNP in gene b	Best SNP p-value d	LD: best SNP in Trios –best SNP in CC [r^2^] c	number of SNPs in gene region
*ESRRA*	0.0616	rs2429455§	0.0065	0.403	12	0.3778	rs1059440§	0.0719	10	0.3853	rs11231740§	0.0681	0.129	11	0.1662	rs4930702&	0.0161	0.004	12
*ESRRG*	0.4240	rs2185226#	0.0035	0.001	335	0.8585	rs12033461#	0.0155	316	0.9301	rs11577585#	0.0185	0	320	0.8458	rs7531250#	0.0090	0.003	349
*GABPA*	0.0400	rs2051180§	0.0022	0.945	36	0.7873	rs11087972§	0.1216	35	0.0261	rs7284014§	0.0012	0.024	32	0.1191	rs2829866§	0.0048	0.206	37
*GABPB1*	0.3542	rs4775886§	0.0205	0	37	0.9953	rs12910368#	0.3374	32	0.1336	rs16963477&	0.0071	0	35	0.1091	rs16963477&	0.0046	0	38
*GABPB2*	0.9071	rs3754210§	0.2138	0.072	19	**0.0644**	**rs4970989** §	**0.0052**	**19**	0.4848	rs267738§	0.0539	NA	19	0.6166	rs7526955§	0.0666	0.243	22
*MEF2A*	0.1643	rs7173943§	0.0057	0.001	59	0.4216	rs4313794§	0.0185	60	0.4036	rs7175248§	0.0156	0.065	59	0.1462	rs7173943§	0.0039	0.001	65
*MYC*	0.2750	rs11990827§	0.0094	0.001	60	0.6785	rs4395860&	0.0422	55	0.4720	rs13252644§	0.0194	0	59	0.9125	rs12155669&	0.0923	0.384	62
*NRF1*	0.2379	rs2693737&	0.0104	0.013	51	0.9805	rs9792084§	0.1758	52	0.6346	rs11771549§	0.0418	0.012	47	0.8021	rs11771549§	0.0756	0.012	56
*NRIP1*	0.1075	rs2776043#	0.0052	0.005	47	0.6005	rs17274722&	0.0506	46	**0.0078**	**rs10482862** &	**0.0003**	**0.004**	**43**	0.1730	rs10482862&	0.0047	0.004	49
*PPARA*	0.6169	rs3744749§	0.0358	0.009	55	0.1999	rs12170325§	0.0084	46	0.2128	rs4253754#	0.0084	0.008	48	0.4980	rs4253655#	0.0219	0.021	55
*PPARD*	0.1854	rs9658085#	0.0097	0.016	40	0.8156	rs2894401&	0.1644	22	0.3453	rs2267666#	0.0201	0.134	35	0.3595	rs9658085#	0.0181	0.016	40
*PPARGC1A*	0.1456	rs17574213*	0.0034	0.001	75	0.8988	rs10517032§	0.0641	71	0.4964	rs17576576§	0.0163	0.009	67	0.5493	rs7682906§	0.0182	0.089	78
*PPARGC1B*	0.3352	rs10069462§	0.0081	0.017	110	0.4494	rs7713955§	0.0141	102	0.5462	rs10065816§	0.0166	0.145	104	0.5180	rs11746690#	0.0096	0.016	114
*SIRT1*	**0.0104**	**rs7895833** f,§	**0.0011**	**0.304**	**16**	0.1282	rs10509291§	0.0190	16	0.0471	rs16924888 f,§	0.0053	0.01	15	**0.0209**	**rs17712705** §	**0.0019**	**0.146**	**17**
*SP1*	0.0964	rs4759082§	0.0126	same SNP	16	0.1949	rs4759082§	0.0436	11	0.1624	rs2016266§	0.0207	0.209	14	0.1173	rs4759082§	0.0101	same SNP	16
*YY1*	0.0470	rs8007801§	0.0034	0.011	25	0.1156	rs9291&	0.0079	26	0.5759	rs2766692§	0.0732	0.015	25	0.1391	rs9291&	0.0085	same SNP	26

- Table 1 will be continued –.

- Table 1 continued -.

a BMI ≥30 (cases) vs. BMI <25 (controls);

b Location of SNP: *, exonic;

# ,intronic;

§ ,upstream of gene and.

& ,downstream of gene;

c Linkage Disequilibrium (LD) was calculated in the parents of the family-based GWAS sample by use of HaploView 4.2;

d SNP-wise p-values of the meta-analysis were derived by application of the METAL software (for details see ‘Materials and Methods, Meta-analysis’);

f LD between rs7895833 and rs16924888: r^2^ = 0.581; best gene of each sample and the meta-analysis is indicated in bold letter.

*ESRRA*, Estrogen related receptor alpha; *ESRR*G, Estrogen related receptor gamma; *GABPA*, GA-binding protein alpha subunit; *GABPB1*, GA-binding protein beta subunit 1; *GABPB2,* GA-binding protein beta subunit 2; *MEF2A*, Myocyte-specific enhancer factor 2A; *MYC*, Myelocytomatosis viral oncogene homolog (avian); *NRF1*, Nuclear respiratory factor 1; *NRIP1*, Nuclear receptor-interacting protein 1; *PPARA*, Peroxisome proliferator-activated receptor alpha; *PPARD*, Peroxisome proliferator-activated receptor delta; *PPARGC1A*, Peroxisome proliferator-activated receptor gamma coactivator 1 alpha; *PPARGC1B*, Peroxisome proliferator-activated receptor gamma coactivator 1 beta; *SIRT1*, Sirtuin 1; *SP1*, Specificity protein 1; *YY1,* Transcriptional repressor protein YY1.

## Discussion

The gene variants discovered by single-locus-oriented GWAS have explained only about 1.5% of the total BMI variance so far [2]. As GSEA approaches concentrate on the combined effects of several loci to potentially reveal new insight into the genetic impact, we performed a GSEA to analyze if autosomal nuclear-encoded mitochondrial genes are enriched for association signals for obesity. The three mitochondrial gene sets as well as the GSEA and MAGENTA procedure were adopted from Segrè et al. [15]. While Segrè et al. [15] did not find enrichment in association with T2DM and related glycemic traits, we observed enrichment for obesity association signals in the gene set of the 16 regulators of nuclear-encoded mitochondrial genes (gene set 1) in two independent case-control GWAS data sets (total n = 1,834). However, the enrichment was not detectable in a family-based GWAS sample of 705 obesity trios. The enrichment for obesity association signals was found for the 50^th^ percentile, i.e. gene adjusted p-values between ∼0.2 and 0.5. By applying MAGENTA to the discovery sample, we also found enrichment above the 75^th^ percentile (Table 1). This observation was similarly present only in the meta-analysis, but not in any of the confirmation samples individually. Our results support the hypothesis that a GSEA may detect combined association effects of several loci [3]–[7]. None of the above described 16 genes revealed significant association to obesity in a single locus-oriented approach, as none of these genes has been found in the list of 32 BMI loci reported in the latest and largest meta-analysis so far [2].

It was not possible to identify one specific weight associated candidate gene, as in both case-control approaches the SNPs/genes with the lowest p-values differed (Table 3). Our finding underscores that the combined effect of several loci leads to an association with the investigated trait, rather than a single gene of a set.

One limitation of our analysis is that it is based exclusively on autosomal mitochondrial genes. According to the MitoCarta compendium there are 1012 unique mitochondrial genes [13] of which 13 are protein coding genes of mtDNA (1.3% of all mitochondrial genes) and 31 are X-chromosomal (one is X/Y-chromosomal; 3.1% of all mitochondrial genes). As most GWAS primarily focus on autosomal SNPs, genes of mtDNA and sex chromosomes were not included in the analysis of Segrè et al. [15]. For reasons of comparability we also only focused on autosomal mitochondrial genes. However, due to the fact that both mtDNA and sex-chromosomal genes represent less than 5% of all mitochondrial genes, the impact of these genes on the enrichment analysis might be small. Anyhow, variation in the mtDNA has probably more relevant effects on the mitochondrial function than variation in autosomal genes, as for instance mtDNA does not comprise UTRs or introns. However, mtDNA SNPs are not the focus of our GSEA.

Another limitation of our GSEA is that it is based on GWAS data, i.e. common variants. Rare variants, which could have a stronger impact on the investigated trait, are thus hardly addressed in our analyses.

We evaluated the robustness of our results pertaining to the 16 nuclear regulators of mitochondrial function. Besides the leading-edge fraction test several other statistical tests recommended were performed (Wilcoxon-Mann-Whitney-test, Kolmogorov Smirnov test, t-test) to demonstrate independence from the method choice. Although the Kolmogorov Smirnov test of the discovery stage revealed only nominal significance, results of all tests were similar regarding significance within a fixed tested sample and gene set. The MAGENTA software was additionally applied to each single sample in order to maximally guarantee robustness (within each sample) and comparability (between different samples and the meta-analysis) of the results. Apart from a few exceptions, we found high levels of agreement of the p-values determined by both methods (leading-edge fraction test vs. MAGENTA) within a sample (Table 1 & 2).

In addition to the robustness regarding method choice another strength of our study was that we used both case-control and family-based samples. We observed consistent evidence for enrichment in two case-control samples but failed to detect it in the family-based GWAS sample of 705 obesity trios. Possibly the family-based sample was too small for a confirmation. Moreover, if the effect was mainly driven by lean and normal weight subjects, the frequencies of the variants would be very low in the predominantly obese trio parents [34] again resulting in power issues. A third explanation might be genetic heterogeneity including both locus and allelic heterogeneity.

The 16 regulators of nuclear-encoded mitochondrial genes (gene set 1) are transcription factors and/or co-activators (Table 3). Although there are hints of disturbed mitochondria or mitochondrial function among obese individuals [9], [10], from the findings of this GSEA, we cannot conclude if or to what extent these nuclear-encoded regulators of gene set 1 are involved in mitochondrial disturbance. There are mouse models for nine of the 16 genes which showed that knockout (k.o.) or alterations in the expression of these genes are related to leanness or related traits (Table 4). For example, *Nrip1* k.o. mice are viable and morphologically normal, but 15–20% lighter than the wild-type or heterozygous littermates [35]. *Nrf2* ( =  *Gabpa*) k.o. mice are characterized by decreased adipose tissue mass and protected against a high fat diet induced obesity. In addition, *Sirt1* transgenic (knockin) mice were lighter than wild type littermates and had less white adipose tissue per body weight [36]. The enrichment of association signals in gene set 1 supports the findings from the above mentioned animal studies that these genes could be potential candidate genes for obesity/leanness and related traits. Similarly, in a recent GSEA, Vimaleswaren et al. [37] found enrichment of association signals for a gene set of 547 obesity-susceptibility candidate genes in a large meta-analysis of 123,564 individuals [2].

**Table 4:** Animal models (knockout, alterations in the expression and mutations) of the nuclear regulators of mitochondrial genes (gene set 1) in relation to obesity or related traits.

Gene	Phenotype	Reference
*ESRRA*	ERRα^−/−^ mice with reduced body weight and fat mass, and resistance toa high-fat diet-induced obesity	[38]
*ESRRG*	No body weight/body fat associated phenotype	
*GABPA*	= *NRF2*; targeted knock-out (k.o.) of *Nrf2* in mice leads to 20% lower body weightafter *ad libitum* diet compared to wild type littermates, lower adipose tissue mass,smaller adipocytes and protects against weight gain and obesity otherwiseinduced by a high fat diet	[39]
*GABPB1*	No body weight/body fat associated phenotype	
*GABPB2*	No body weight/body fat associated phenotype	
*MEF2A*	No body weight/body fat associated phenotype	
*MYC*	Transgenic mice overexpressing c-*myc* in the liver show lower body weightincrease and lower fat accumulation in adipose tissue compared to controlmice on a high fat diet of 3 months	[40]
*NRF1*	No body weight/body fat associated phenotype	
*NRIP1*	Formerly known as *RIP140*; k.o. mice viable and morphologically normal,but 15–20% less heavier than wild-type or heterozygous littermates	[35]
*PPARA*	*PPARα*-null mice on two different backgrounds (Sv/129 or C57BL/6N) were notobese, but had hepatic accumulation of fat and larger gonadal adipose storescompared to wild type controls	[41]
*PPARD*	*PPARδ*-null mice are smaller than controls and have smaller gonadal fat stores	[42]
*PPARGC1A*	Female PGC-1α^−/−^ mice show increased body fat and hepatic steatosis aftershort term starvation	[43]
*PPARGC1B*	*PGC-1b* k.o. mice with reduced body weight and fat mass	[44]
*SIRT1*	Sirt1 transgenic (knockin) mice are lighter and have less white adipose tissueper body weight than wild type littermates	[36]
*SP1*	No body weight/body fat associated phenotype	
*YY1*	No body weight/body fat associated phenotype	

*ESRRA*, Estrogen related receptor alpha; *ESRR*G, Estrogen related receptor gamma; *GABPA*, GA-binding protein alpha subunit; *GABPB1*, GA-binding protein beta subunit 1; *GABPB2,* GA-binding protein beta subunit 2; *MEF2A*, Myocyte-specific enhancer factor 2A; *MYC*, Myelocytomatosis viral oncogene homolog (avian); *NRF1*, Nuclear respiratory factor 1; *NRIP1*, Nuclear receptor-interacting protein 1; *PPARA*, Peroxisome proliferator-activated receptor alpha; *PPARD*, Peroxisome proliferator-activated receptor delta; *PPARGC1A*, Peroxisome proliferator-activated receptor gamma coactivator 1 alpha; *PPARGC1B*, Peroxisome proliferator-activated receptor gamma coactivator 1 beta; *SIRT1*, Sirtuin 1; *SP1*, Specificity protein 1; *YY1,* Transcriptional repressor protein YY1.

In summary, a GSEA on autosomal nuclear-encoded genes relevant for mitochondrial function revealed that a gene set of 16 nuclear encoded regulators of mitochondrial genes was enriched for weak obesity association signals. Initially, this enrichment was found in a case-control approach and independently confirmed in another case-control sample.

## Supporting Information

Table S1 **Basic phenotypical characteristics of the family-based, the case-control and the population-based GWAS sample.**
(DOC)

Table S2 **Quality control of SNPs.**
(DOC)
