# Supplementary material for: Gene Set of Nuclear-Encoded Mitochondrial Regulators Is Enriched for Common Inherited Variation in Obesity
Source: PLoS One. 2013 Feb 8;8(2):e55884. doi: 10.1371/journal.pone.0055884 (PMC3568071; doi:10.1371/journal.pone.0055884)
Supplement: Table S1 — Basic phenotypical characteristics of the family-based, the case-control and the population-based GWAS sample. (DOC) [file pone.0055884.s001.doc]

**Table S1** **Basic phenotypical characteristics of the family-based, the case-control and the population-based GWAS sample**

| **Sample** | **description** | **status** | **n total** | **age in years ^a^** | **BMI in kg/m^2 a^** | **BMI SDS ^a, b^** |
| --- | --- | --- | --- | --- | --- | --- |
|  |  |  | **{% female}** | **{female}** | **{female}** | **{female}** |
|  |  |  |  | **(male)** | **(male)** | **(male)** |
| **(1) family-based GWAS sample** | (extremely) obese children and adolescents | cases | 705 | 13.44 ± 3.01 | 32.02 ± 5.82 | 4.23 ± 1.96 |
|  |  |  | {54.89} | {13.54 ± 3.04} | {32.36 ± 6.04} | {4.50 ± 2.03} |
|  |  |  |  | (13.31 ± 2.98) | (31.60 ± 5.51) | (3.91 ± 1.81) |
|  | parents of the (extremely) obese children and adolescents | parents | 1410 | 42.54 ± 6.02 | 30.28 ± 6.33 | 1.65 ± 1.84 |
|  |  |  | {50.00} | {40.89 ± 5.44} | {30.23 ± 7.12} | {1.75 ± 1.88} |
|  |  |  |  | (44.21 ± 6.12) | (30.34 ± 5.42) | (1.55 ± 1.78) |
| **(2) case-control GWAS sample** | (extremely) obese children and adolescents | cases | 453 | 14.37 ± 3.75 | 33.15 ± 6.68 | 4.55 ± 2.16 |
|  |  |  | {58.00} | {14.50 ± 3.67} | {33.18 ± 6.84} | {4.66 ± 2.18} |
|  |  |  |  | (14.18 ± 3.85) | (33.11 ± 6.48) | (4.40 ± 2.12) |
|  | lean or normal weight subjects | controls | 435 | 26.08 ± 5.75 | 18.31 ± 1.11 | -1.45 ± 0.34 |
|  |  |  | {61.00} | {26.54 ± 6.37} | {17.58 ± 0.95} | {-1.35 ± 0.30} |
|  |  |  |  | (25.38 ± 4.57) | (18.86 ± 0.94) | (-1.60 ± 0.36) |
| **(3) Population-based GWAS sample (KORA)** |  |  |  |  |  |  |
| **(3a) Full sample** | population-based | population-based | 1743 | 53.87 ± 8.86 | 27.75 ± 4.56 | 0.68 ± 1.28 |
|  |  |  | {51.06} | {53.63 ± 8.80} | {27.51 ± 5.08} | {0.71 ± 1.30} |
|  |  |  |  | (54.12 ± 8.91) | (28.00 ± 3.94) | (0.65 ± 1.27) |
| **(3b) As case-control sample** | obese subjects (BMI ≥ 30) | Cases | 463 | 55.84 ± 8.54 | 33.55 ± 3.66 | 2.31 ± 1.10 |
|  |  |  | {53.13} | {56.46 ± 8.36} | {34.04 ± 3.75} | {2.37 ± 1.00} |
|  |  |  |  | (55.14 ± 8.70) | (33.00 ± 3.48) | (2.24 ± 1.19) |
|  | normal weight subjects (BMI < 25) | Controls | 483 | 50.55 ± 8.64 | 22.84 ± 1.48 | -0.61 ± 0.45 |
|  |  |  | {66.25} | {49.83 ± 8.17} | {22.63 ± 1.51} | {-0.49 ± 0.41} |
|  |  |  |  | (51.96 ± 9.38) | (23.26 ± 1.32) | (-0.83 ± 0.42) |

^a^ Data is shown as mean ± standard deviation.

^b^ Age and BMI SDS (standard deviation score) values were available for 693 mothers and 686 fathers of the family-based GWAS sample. Calculation of the BMI SDS values has been based on population reference values following the National Nutrition Survey I [24].
