# Supplementary material for: Gene Set of Nuclear-Encoded Mitochondrial Regulators Is Enriched for Common Inherited Variation in Obesity
Source: PLoS One. 2013 Feb 8;8(2):e55884. doi: 10.1371/journal.pone.0055884 (PMC3568071; doi:10.1371/journal.pone.0055884)
Supplement: Table S2 — Quality control of SNPs. (DOC) [file pone.0055884.s002.doc]

**Table S2** **Quality control of SNPs**

| **Sample** | **KORA** | **TRIOs** | **CCs** |
| --- | --- | --- | --- |
|  | (n=1,743) | (705 trios; n=2,115) | (453 cases vs.435 controls; n=888) |
| **number of autosomal SNPs genotyped ^a^** | 868,278 | 868,257 | 869,224 |
| **1^st^ SNP-QC criterion** | sample call-rate per SNP ≥ 95% | | |
| **number of SNPs failing 1^st^ SNP-QC criterion** | 79,032 | 36,507 | 33,616 |
| **% of SNPs failing 1^st^ SNP-QC criterion** | 9.10% | 4.20% | 3.87% |
| **2^nd^ SNP-QC criterion** | MAF ≥ 1% | MAF ≥ 5% | MAF ≥ 1% |
|  | in sample | in parents | in sample |
| **number of SNPs failing 2^nd^ SNP-QC criterion** | 120,498 | 192,243 | 134,847 |
| **% of SNPs failing 2^nd^ SNP-QC criterion** | 13.88% | 22.14% | 15.51% |
| **3^rd^ SNP-QC criterion** | two-tailed exact p-value in test for HWE ≥ 0.001 | | |
|  | in sample | in parents (after exclusion of Mendelian incorrect genotype calls) | in controls |
| **number of SNPs failing 3^rd^ SNP-QC criterion** | 40,538 | 13,050 | 4,563 |
| **% of SNPs failing 3^rd^ SNP-QC criterion** | 4.67% | 1.50% | 0.52% |
| **4^th^ SNP-QC criterion** | none | at least one minor and one major allele transmission | none |
| **number of SNPs failing 4^th^ SNP-QC criterion** | 0 | 67,682 | 0 |
| **% of SNPs failing 4^th^ SNP-QC criterion** | 0.00% | 7.80% | 0.00% |
| **number of SNPs left after SNP-QC ^b^** | 659,502 | 641,991 | 703,015 |
| **% of SNPs left after SNP-QC ^b^** | 75.96% | 73.94% | 80.88% |

^a^ This number is differing due to the fact that each sample has been analyzed at different points of time and for each moment of analysis the latest Affymetrix annotation file has been used.

^b^ There are SNPs failing more than one SNP-QC criterion.
